# Neoadjuvant lenvatinib in a patient with invasive poorly differentiated thyroid cancer

**DOI:** 10.3332/ecancer.2025.1902

**Published:** 2025-04-25

**Authors:** Santiago Zund, Inés Califano, Fernando Carrizo, Sergio Quildrian

**Affiliations:** 1 Department of Head and Neck Surgery, Instituto de Oncología Ángel H. Roffo (IOAHR), University of Buenos Aires, Buenos Aires C1417DTB, CABA, Argentina; 2 Department of Endocrinology, Instituto de Oncología Ángel H. Roffo (IOAHR), University of Buenos Aires, Buenos Aires C1417DTB, CABA, Argentina; 3 Department of Pathology, Instituto de Oncología Ángel H. Roffo (IOAHR), University of Buenos Aires, Buenos Aires C1417DTB, CABA, Argentina; 4 Department of Soft Tissue Tumors, Instituto de Oncología Ángel H. Roffo (IOAHR), University of Buenos Aires, Buenos Aires C1417DTB, CABA, Argentina; a https://orcid.org/0000-0001-6357-6136

**Keywords:** thyroid cancer, lenvatinib, tyrosine kinase inhibitors, poorly differentiated, neoadjuvant

## Abstract

**Background:**

A limited number of case reports have investigated the role of tyrosine kinase inhibitors as neoadjuvant therapy in locally invasive poorly differentiated thyroid cancer (PDTC).

**Case report:**

A 69-year-old female patient presented with a large mass in the right thyroid lobe measuring 10 × 8 cm. A computed tomography scan showed a mass with no cleavage plane between the tumour and both the laryngotracheal and esophageal right lateral wall. A core needle biopsy was performed and confirmed PDTC. The case was considered unresectable. After a trial of neoadjuvant lenvatinib was administered, a partial response of 50% was achieved and surgery could be performed with favourable surgical outcomes. The patient did not resume lenvatinib after surgery, as no evidence of structural disease was found. At publication, she remains free of structural disease with an incomplete biochemical response.

**Conclusion:**

Neadjuvant lenvatinib can be a therapeutic option to reduce primary tumour extent, in order to perform surgery and achieve better outcomes. However, this requires a highly selected patient group. Neoadjuvant-targeted therapy holds significant promise and warrants further investigation.

## Introduction

Poorly differentiated thyroid cancer (PDTC) is a rare and aggressive form of follicular-cell-derived thyroid carcinoma, accounting for 3%–5% of all thyroid tumours. In 2007, diagnostic criteria called the Turin proposal, were advocated by an international consensus group. The Turin proposal requires three criteria: 1) solid/trabecular/insular growth pattern, 2) absence of nuclear features of papillary carcinoma and 3) at least one of the following three features: mitotic index ≥3/10 high power fields, necrosis or convoluted nuclei [1]. This tumour shows intermediate characteristics and behaviour between the indolent differentiated thyroid cancer (DTC) and the rapidly growing, often fatal anaplastic thyroid cancer (ATC), with a poor overall prognosis.

The majority of patients present with advanced locoregional or metastatic disease [2, 3]. Surgery remains the mainstay of treatment; however, it rarely achieves complete resection and it is usually associated with significant morbidity [4].

Lenvatinib is a tyrosine kinase inhibitor (TKI) that has demonstrated objective response rates of 64.8% and improved progression-free survival in patients with radioactive iodine (RAI) refractory progressive DTC [5]. There are some reports of its use with neoadjuvant purposes in DTC [6]. It has been used in a few cases as neoadjuvant therapy in PDTC [7–9].

We report a case of a patient with PDTC who was firstly deemed unresectable due to local extent and comorbidities, but later successfully operated on with negative margins after lenvatinib neoadjuvant therapy.

## Case report

**Figure 1.**
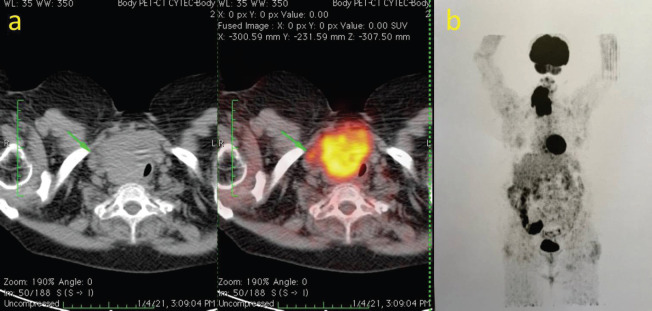
(a): CT scan and ^18^.FDG.PET.CT scan, at initial presentation (axial). (b): ^18^.FDG.PET scan, coronal view.

A 69-year-old female patient with a record of diabetes mellitus, hypertension, chronic asthma and a history of a right benign nodular goiter of 5 years, presented with sudden enlargement of the thyroid mass. Due to the SARS-Cov2 pandemic, she did not seek consultation for 7 months. By this time, she presented with dyspnea, permanent cough, hoarseness and dysphagia. Clinical examination revealed a locally advanced right thyroid mass, deeply fixed and immobile and a right vocal cord paralysis. A computed tomography (CT) scan showed a large mass in the right thyroid lobe measuring 10 × 8 × 6 cm, with calcifications and areas of necrosis. There was no cleavage plane between the tumour and both the laryngotracheal and esophageal right lateral wall. She also underwent a fluorodeoxyglucose-positron emission tomography (FDG. PET) that showed a highly FDG avid tumour (SUV max 11.6) and did not show pathologic uptake outside the thyroid (Figure 1).

Due to the impossibility of accurately identifying the histotype with physical examination, imaging basis and fine needle aspiration biopsy, a percutaneous core needle biopsy was performed to rule out ATC, lymphoma, IgG4 thyroiditis and PDTC. All morphological findings were consistent with PDTC. The immunohistochemistry panel was positive for p53 and CK7, focally positive for thyroglobulin (Tg) and TTF-1 and negative for calcitonin, with a Ki-67 proliferation index of 10%.

**Figure 2.**
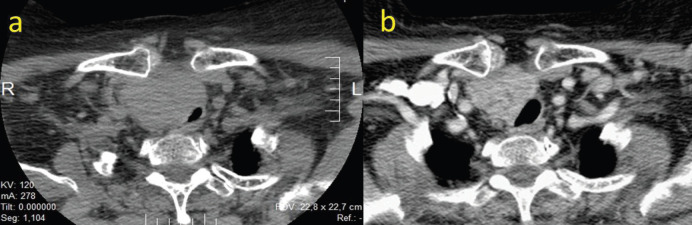
(a): CT scan of the neck pre-neoadjuvant therapy. (b): CT scan after 10 weeks of lenvatinib treatment (24 mg/day).

The case was presented in the head and neck tumour board (H&NTB), where it was deemed that a complete surgical resection was unlikely, and morbidity would be unacceptably high. The consensus was to attempt a neoadjuvant trial with lenvatinib. The patient began therapy with lenvatinib 24 mg once daily. After 10 weeks of therapy, she experienced an improvement of the compressive symptoms, with no significant side effects, except for grade I fatigue, grade I hypertension and a grade II hand-foot-skin-reaction (HFSR). Enhanced neck CT scan objectivised a tumour measuring 7 × 5, 5 × 5 cm, a volumetric decrease of more than 50% of the cervical tumour, with a reduction in the mass effect on the larynx, trachea and the esophagus. An image highly suspicious of a tumour embolus was evident in the right internal jugular vein (Figure 2).

**Figure 3.**
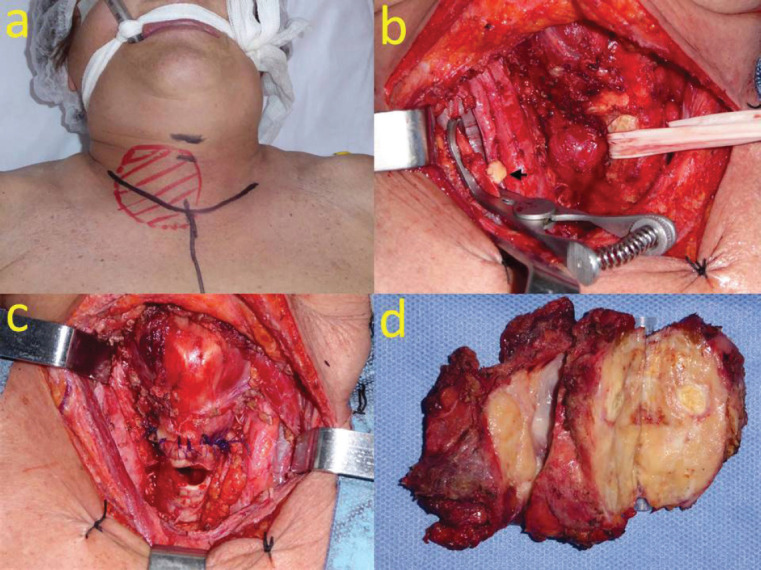
(a): Surgical approach. (b): Embolectomy of the right internal jugular vein (arrow: tumoural emboli). (c): Surgical field after total thyroidectomy, tracheal sleeve resection and end-to-end anastomosis. (d): Surgical specimen.

After discussing the case in the H&NTB, it was considered that due to the objective reduction of the cervical mass, surgery was feasible. Lenvatinib was discontinued 10 days before surgery, after a neoadjuvant trial of 22 weeks. The patient underwent total thyroidectomy with *en-bloc* resection of strap muscles, right recurrent laryngeal nerve, tracheal sleeve resection of four rings with end-to-end anastomosis, embolectomy of the right internal jugular vein and a transitory tracheostomy to protect tracheal anastomosis. As there was no gross invasion of the larynx and esophagus, a shave and a muscular resection on these structures were performed, respectively. There was no need for a sternotomy approach (Figure 3).

Postoperatively, histopathology report yielded a PDTC of 8 cm in largest diameter, extensive vascular invasion, tracheal infiltration, a 1 cm PDTC embolus in the right jugular vein, 2 negative perithyroidal lymph nodes and negative margins (pT4a pN0 M0 E IVa).

No airway complications occurred postoperatively; however, hypocalcemia was found. After 2 months, a CT scan was performed with no evidence of structural disease. She received 100 mCi of ^131^I with uptake exclusively in the neck; the preablation Tg level was 14.5 ng/mL, with negative antithyroglobulin (anti-Tg) antibodies. Lenvatinib was not resumed due to a lack of structural disease.

After repeated neck, chest, abdomen and pelvis CT performed 6, 14, 24, 30 and 38 months after surgery, no structural disease was found. An incomplete biochemical response was noted (Tg 11, 4.1, 2.8, 3.6 and 4.7 ng/mL, respectively, with negative anti-Tg antibodies). The patient remains alive and free of structural disease after 40 months and continues in clinical follow-up.

## Discussion

Locally advanced management of PDTC is controversial and depends on the extent of invasion of critical structures in the neck, the presence of distant metastasis and patient-related factors. Clinically, these tumours occur in older patients, with a slight female preponderance and develop as rapidly growing masses (4 cm or more). Surgical resection in these patients may be deemed impossible or inappropriate and can also be associated with significant morbidity. Historically, limited options were available for these cases [10].

Lenvatinib is an oral multi-kinase inhibitor drug that targets vascular endothelial growth factor receptors 1–3, fibroblast growth factor receptors 1–4, RET, c-kit and platelet-derived growth factor receptor α. Its anti-angiogenic and anti-tumoural activity in RAI-refractory thyroid cancer has been clearly demonstrated in the SELECT trial, and it was approved by the Food and Drug Administration in 2016 [5]. The efficacy of lenvatinib in the treatment of progressive disease suggested an opportunity for its use in the neoadjuvant setting. It has been reported that lenvatinib shrinks the tumour size in order to facilitate ensuing surgery and to spare the patient from the morbidities of a more aggressive resection. The positive effect has also been noted and reported in metastatic disease [11].

Very limited experience exists with neoadjuvant therapy with lenvatinib in PDTC. Only 3 cases deemed unresectable have been published so far. Gay *et al* [7] presented an 81-year-old woman with a PDTC, that initially was treated with external beam radiation (20 Gy) and later with lenvatinib 10 mg/day with neoadjuvant intent. After 2 months of therapy, she was operated successfully and received 150 mCi of RAI. A whole-body scan showed mild uptake in the neck, without signals of recurrent or metastatic iodine avid disease [7]. Molinaro *et al* [8] published a case of a 62-year-old female with a recurrent PDTC locally invasive in trachea, with no cleavage plane with the cricoid cartilage and the esophagus. Lenvatinib therapy was administered at a dose of 20 mg/day via a nasogastric tube. Unfortunately, the patient developed a double esophageal perforation and died one month later from a pulmonary embolism [8]. Alshehri *et al* [9] reported a 56-year-old woman with a T4aN0M1 PDTC, with no fat plane between the mass, the trachea and esophagus. The patient started a sorafenib therapy trial (400 mg/day), but was stopped within a week due to body pain and fatigue. She immediately commenced on lenvatinib 10 mg/day, and after 2 months, a CT scan showed and interval reduction in the size and mass effect on the trachea. She underwent complete excision of the tumour with no tracheal resection. Three months later, she received 200 mCi of RAI therapy, with uptake in the thyroid bed, the lung and the sterni manubrium [9].

Common adverse events related to lenvatinib include hypertension, asthenia, fatigue, rash, proteinuria, HFSR and diarrhea. Additionally, as a powerful antiangiogenic drug, lenvatinib can cause fistula formation or can lead to mucosal perforation of the trachea and/or esophagus, especially in locally invasive cancer. Due to this antiangiogenic activity, it is recommended that lenvatinib should be discontinued 6 days prior to surgery [12]. In our patient, lenvatinib was used at full dose for 22 weeks; it was relatively well tolerated and led to a significant reduction of tumour size. The drug was discontinued 10 days before surgery and no relevant post-operative complications were found.

Clinical trials evaluating the neoadjuvant use of multi-kinase inhibitors are in progress around the world [13]. Further investigation is needed regarding neoadjuvant therapy in locally invasive DTC and PDTC. Increasing experience of indications, optimum dosing and administration length, are likely to improve patient outcomes [14].

## Conclusion

In summary, we report a case of PDTC, initially considered inoperable, that was fully resected after a preoperative neoadjuvant trial with TKIs. Neadjuvant lenvatinib was a feasible therapeutic option in our selected case. It was effective in reducing tumour size, in order to facilitate and spare the patient from the morbidities of a more aggressive resection. More research and information are expected to establish evidence-based neoadjuvant therapy guidelines in thyroid cancer in the near future.

## Conflicts of interest

There are no conflicts of interest to declare in this publication.

## Funding

The listed authors did not receive funding for preparing this manuscript.

## Informed consent

The patient´s prior informed consent was obtained for this case report.

## Author contributions

S.Z.: Writing-original draft preparation, writing-review and editing.

I.C.: Writing-review and editing.

F.C.: Writing-review and editing.

S.Q.: Writing-review and editing.

## References

[1] Volante M, Collini P, Nikiforov Y (2007). Poorly differentiated thyroid carcinoma: the Turin proposal for the use of uniform diagnostic criteria and an algorithmic diagnostic approach. Am J Surg Pathol.

[2] Haugen BR, Alexander EK, Bible K (2016). 2015 American Thyroid Association Management Guidelines for adult patients with thyroid nodules and differentiated thyroid cancer. Thyroid.

[3] Baloch ZW, Asa SL, Barletta JA (2022). Overview of the 2022 WHO classification of thyroid neoplasms. Endocr Pathol.

[4] Shindo ML, Caruana SM, Kandil E (2014). Management of invasive well-differentiated thyroid cancer: an American Head and Neck Society consensus statement. Head Neck.

[5] Schlumberger M, Tahara M, Wirth LJ (2015). Lenvatinib versus placebo in radioiodine-refractory thyroid cancer. N Engl J Med.

[6] Yeo JJY, Stewart K, Maniam P (2023). Neoadjuvant tyrosine kinase inhibitor therapy in locally advanced differentiated thyroid cancer: a single centre case series. J Laryngol Otol.

[7] Gay S, Monti E, Antonelli CT (2019). Case report: lenvatinib in neoadjuvant setting in a patient affected by invasive poorly differentiated thyroid carcinoma. Future Oncol.

[8] Molinaro E, Viola D, Viola N (2019). Lenvatinib administered via nasogastric tube in poorly differentiated thyroid cancer. Case Rep Endocrinol.

[9] Alshehri K, Alqurashi Y, Merdad M (2022). Neoadjuvant lenvatinib for inoperable thyroid cancer: a case report and literature review. Cancer Rep.

[10] Stewart KE, Strachan MWJ, Srinivasan D (2019). Tyrosine kinase inhibitor therapy in locally advanced differentiated thyroid cancer: a case report. Eur Thyroid J.

[11] Masaki C, Sugino K, Saito N (2017). Lenvatinib induces early tumor shrinkage in patients with advanced thyroid carcinoma. Endocr J.

[12] Hartl D, Guerlain J, Bresuskin I (2020). Surgery in the context of kinase inhibitor therapy for locally invasive thyroid cancer. Eur J Surg Oncol.

[13] Shonka DC, Ho A, Chintakuntlawar AV (2022). American Head and Neck Society Endocrine Surgery Section and International Thyroid Oncology Group consensus statement on mutational testing in thyroid cancer: defining advanced thyroid cancer and its targeted treatment. Head Neck.

[14] Rusell MD, Kamani D, Randolph GW (2020). Modern surgery for advanced thyroid cancer: a tailored approach. Gland Surg.

